# Bone health perspectives among Indigenous people: a qualitative study

**DOI:** 10.5694/mja2.52704

**Published:** 2025-06-17

**Authors:** Troy Walker (Yorta Yorta), Karan P Singh, Vanessa Gan, Brooke Conley (Ngiyampaa), Jessica Bravo, Nigel Smith (Weilwan), April Clarke (Eastern Maar, Kirrae Whurrung, Djap Wurrung), Jackson Baker, Louise J Maple‐Brown, Robin M Daly, Jennifer Browne, Jesse Zanker, Cat Shore‐Lorenti, David Scott, Peter R Ebeling, Ayse Zengin

**Affiliations:** ^1^ Monash University Melbourne VIC; ^2^ National Centre for Healthy Ageing Melbourne VIC; ^3^ University of Melbourne Melbourne VIC; ^4^ Victorian Aboriginal Community Controlled Health Organisation Melbourne VIC; ^5^ Murrumbidgee Local Health District Moama NSW; ^6^ Gariwerd Dreaming Melbourne VIC; ^7^ Njernda Aboriginal Corporation Echuca VIC; ^8^ Menzies School of Health Research Charles Darwin University Darwin NT; ^9^ Royal Darwin and Palmerston Hospitals NT Health Darwin NT; ^10^ Institute for Physical Activity and Nutrition, Deakin University Melbourne VIC; ^11^ Deakin University Melbourne VIC; ^12^ Royal Melbourne Hospital Melbourne VIC

**Keywords:** Education, public health, Aging, Health communication, Health promotion, Fractures, bone, Musculoskeletal pain, Bone, Osteoporosis

## Abstract

**Objectives:**

To explore perspectives and beliefs on bone health among Indigenous adults in Victoria.

**Design:**

Qualitative focus groups with semi‐structured questions. Focus group discussions were analysed for themes and subthemes using an Indigenous research framework based on three concepts: Ways of Knowing, Ways of Being and Ways of Doing.

**Setting, participants:**

Focus groups were conducted at Aboriginal Community‐controlled organisations and Community centres. Men and women aged ≥ 35 years who identified as Indigenous and were able to give informed consent were invited to participate.

**Results:**

Eighty‐two Indigenous people participated in twelve focus groups across ten sites in Victoria. Most participants (64) were women, and the majority lived in metropolitan centres, regional centres and large rural towns (Modified Monash categories 1–3). Five themes were developed around the Indigenous framework proposed by Karen Martin‐Booran Mirraboopa — Ways of Knowing, Ways of Doing and Ways of Being — which guided participants in identifying knowledge of exercise for bone and muscle health; connection to Country; importance of regular preventive health activities; food and nutrients as good medicine for bone health; and healthy futures for Community through education. An overarching theme of holistic health, including the aspect of spirituality and related lifestyle factors pertaining to musculoskeletal health, was highlighted.

**Conclusion:**

Increasing bone health awareness by a co‐created Community education program was valued as it would be beneficial for Indigenous people across the life course. To be effective, incorporating traditional Indigenous ways and knowledge along with present‐day health evidence is required.

**The known**: There is a higher prevalence of bone‐related conditions in Indigenous Australians than in non‐Indigenous Australians.

**The new**: Several considerations to optimise bone health were identified, demonstrating the need for a more holistic approach, as key components of spirituality and co‐created educational programs are often neglected in Indigenous people's health.

**The implications**: A Community educational program co‐created with Indigenous people may increase bone health knowledge. Furthermore, the impact of chronic disease detrimentally affecting bone health of Indigenous people merits more attention and increased financial support.

Bone health is an under‐researched area, with scarce data available in the Indigenous population.^1^, ^2^ Indigenous Australians have a greater fracture risk than non‐Indigenous Australians. Indigenous men and women are 50% and 26%, respectively, more likely to experience fragility fractures (fractures resulting from trauma equal to or less than a fall from standing height) compared with non‐Indigenous Australians.^3^ Hip fractures occur at younger ages in Indigenous Australians compared with non‐Indigenous Australians: 65 versus 81 years in men and 74 versus 83 years in women.^3^ Between 1999 and 2009, age‐related hip fracture rates increased by 7.2% per year for Indigenous adults, but decreased by 3.4% per year for non‐Indigenous adults.^4^ Fragility fractures in older people are associated with increased risk of subsequent fracture and premature mortality.^5^, ^6^

Prevalence of chronic disease, such as cardiovascular disease, type 2 diabetes and kidney disease, is higher among Indigenous adults and associated with increased risk of osteoporosis, falls and fracture.^1^ Despite the impact of falls on Indigenous health, current policy lacks focus on bone conditions, and pain is often a pertinent finding to establish underlying fracture and disease.

Understanding perspectives and beliefs about bone health in the Indigenous population is essential for designing effective, culturally safe programs and services. Sparse data exist about knowledge, attitudes and service preferences relating to bone health among Indigenous people. In this study, we aimed to explore the perspectives, beliefs and knowledge on bone health among Indigenous adults in Victoria, and topics considered essential to increase Community health literacy by co‐creating an educational program for Community members.

## Methods

We report this qualitative study according to the CONSIDER statement criteria for strengthening reporting of health research involving Indigenous peoples (Supporting Information, box 1).^7^ Definitions of the terms Community, Yarning and Positionality, as used in this article, are provided in Box 1.

Box 1 **Definitions of terms used in this article**

**Table:** 

Term	Definition
Community	In this article, Community is capitalised to reflect the Aboriginal and Torres Strait Islander people as a single individual or as a collective group of people. It can also be considered to define a health organisation owned and operated by Aboriginal and Torres Strait Islander people such as with an ACCHO.^8^
Yarning	Yarning is a form of conversation had by Aboriginal and Torres Strait Islander people. It takes on many forms in research literature but it often simply denotes that two or more people get together to engage in a conversation collaboratively.^9^, ^10^, ^11^
Positionality	Positionality is the worldview, perspectives and reflections of the researcher(s). We acknowledge our positionality as Aboriginal and non‐Indigenous researchers. In particular as Aboriginal researchers, clinicians and Community workers, we pedestalise the importance of reflexivity in this approach to working with our ACCHO members as best practice and in taking a strength‐based approach throughout the project.^12^

ACCHO = Aboriginal Community Controlled Health Organisation.

### Study governance, Community engagement and recruitment

This study was led by an Aboriginal researcher (TW) and a bone researcher (AZ) who worked with a team of Indigenous health workers within their respective Aboriginal Community Controlled Health Organisations (ACCHOs) with expertise in Community liaison, partnership building, governance, participation and Elder care. Ongoing collaboration came from the establishment of an Indigenous advisory group (AC, BC, JB, NS and JB) who cross‐checked methodological approaches via consultations with TW and AZ. Our positionality statements are available below.

Invitations for participation were distributed to ACCHOs and Community members throughout Victoria with online and printed flyers, classified by Modified Monash Model geographical location. Inclusion criteria required that participants were aged ≥ 35 years, identified as Indigenous, and were able to give informed consent. The age criterion of ≥ 35 years was selected based on bone breakdown with increased falls risk following the third decade of life.^13^

### Framework

The Indigenous framework proposed by Karen Martin‐Booran Mirraboopa (educator and scholar) was applied and is grounded on three concepts: Ways of Knowing, Ways of Being and Ways of Doing.^14^ The three concepts work in a continuous cycle: Ways of Knowing guide Ways of Being and Ways of Being underpin Ways of Doing, and this facilitates learning and traverses back into enhanced Ways of Knowing (Box 2).

Box 2 **Themes and subthemes raised on Indigenous framework proposed by Karen Martin**

**Table:** 

Framework domain^14^	Theme	Subthemes
Ways of Knowing: the process of listening, exchanging, sharing and engaging	Knowledge of exercise for bone and muscle health	Relevant exercise practice for osteoporosis and bone health
		The right exercise for the right system
Ways of Knowing: the process of listening, exchanging, sharing and engaging	Health prevention through screening and Community awareness	The role of allied health in person‐centred care
		The importance of lifestyle, regular health checks and chronic disease
		Time constraints and cultural awareness
Ways of Doing: experience of life stage, gender, practice, and protection	Connection to Country	Traditional knowledge systems in health outcomes
		Distrust of medical and western conventions
Ways of Doing: experience of life stage, gender, practice, and protection	Food and nutrients as good medicine for bone health	Healthy foods first and supplements second
		Self‐determined nutrition through Community dialogue
		Urbanisation and colonialism impacting food choice
		Cost, access and availability of healthy food
Ways of Being: existing in a network or reciprocal relations and custodianship	Healthy futures for Community through education	Focus on youth
		Focus on Elders
		Visual learning aids
		Keep it local

### Data collection

We conducted 12 face‐to‐face focus groups across metropolitan, rural and regional Victoria during the period 1 April 2021 to 30 September 2023. Two of the 12 focus groups were held during the period 1 April 2021 to 30 June 2021, before the study was paused due to the coronavirus disease 2019 (COVID‐19) pandemic, and the remainder were conducted when the study restarted in 2023. Focus groups were held in roundtable yarning format with at least six Indigenous participants, overseen by Indigenous team members.^9^ Semi‐structured questions were co‐developed by Indigenous team members (Supporting Information, box 2).

Focus groups were conducted with an Aboriginal researcher (TW) and bone researcher (AZ), with assistance from a third bone researcher (CSL). Yarning, an Indigenous research method and cultural form of conversation,^10^, ^11^ allowed researchers and participants to establish trust, and have discussions in a familiar, culturally safe environment. To facilitate this, TW started with an introduction, and this was followed by participant introductions. Subsequently, focus groups followed a semi‐structured question guide with three sections. The first section focused on bone health and explored participants’ knowledge about osteoporosis and maintaining healthy bones. The second section explored knowledge and perspectives of other conditions that affect bone health; for example, cardiovascular disease, chronic kidney disease and type 2 diabetes. The third section discussed how participants preferred the delivery of educational content. Focus groups were audio‐recorded and transcribed using artificial intelligence transcription service Otter (Otter.ai); transcriptions were checked by one of us (KPS), making corrections where necessary. Field notes were written to aid analysis.

### Data analysis

Transcripts were coded in NVivo 11 (QSR International). The data were inductively analysed by two of us (TW and KPS) using thematic analysis approach noting past incorporation with qualitative Indigenous health research.^15^ Audio recordings and transcripts were listened to, read and categorised. Themes generation was discussed by two of us (TW and KPS) and one of us (AZ) confirmed consensus agreement. Preliminary themes were summarised and presented to our Indigenous advisory group for review; feedback was incorporated into the final set of themes, and subthemes were generated from the data using an inductive approach mapped on the Indigenous framework.^14^

### Positionality statement

Troy Walker, is a proud Yorta Yorta (Wollithiga, Yalaba Yalaba and Moira) man with Greek, English and Balkan ancestry. He was born and raised, and is working and living on Yorta Yorta Country; he is a chiropractor holding a Masters in Nutrition and a Clinical Fellow in Lifestyle Medicine. He has clinical, teaching and research expertise in musculoskeletal health, nutrition and public health and strength‐based approaches to the interface between Aboriginal research and health practice. He has worked locally and beyond with his Community as well as within both state and national contexts and he has a particular focus and passion on preventive health, falls risk and the use of lifestyle interventions to facilitate the health of Aboriginal people.

Brooke Conley, a proud Ngiyampaa woman from Cobar, New South Wales, is a physiotherapist and post‐doctoral researcher based in Naarm on Wurundjeri Country. She has research expertise in musculoskeletal conditions, with a particular focus on improving outcomes and care for Aboriginal and Torres Strait Islander peoples.

Nigel Smith is a Weilwan man, living and working on Yorta Yorta Country. Nigel has worked in the fields of mental health and drug and alcohol for over 20 years, 14 of which have been with Murrumbidgee Local Health District. During that time, he has worked in clinical, operational and strategic roles, and his current role is Mental Health Drug and Alcohol Services Coordinator for Aboriginal Peoples. He is passionate about empowering First Nations people to lead a path forward for all people to have a balanced and contemporary understanding of mental health and wellbeing.

Professor Louise Maple‐Brown is Deputy Director (Research) at Menzies School of Health Research and a Senior Endocrinologist at Royal Darwin Hospital. Louise is the senior diabetes researcher at Menzies, having established and led for 11 years the Diabetes across the Lifecourse: Northern Australian Partnership. She has lived and worked in Darwin for over 20 years.

Ayse Zengin is a non‐Indigenous researcher working with various ethnic populations, including Aboriginal and Torres Strait Islander people. She comes from a Turkish immigrant family and is the first university graduate in her family. She was born and raised on the regional south coast of NSW, Australia (Wollongong), of the Dharawal people. Ayse has worked with various Aboriginal and Torres Strait Islander Communities for over eight years. Her main focus is bone health in these populations to improve screening, diagnosis and access to treatment for osteoporosis, as well as increasing health literacy regarding musculoskeletal health.

Karan Partap Singh is a non‐Indigenous third‐year medical student studying at Monash University. He was born in India, but his family moved to Australia when he was three years old, and he has been brought up in Victoria ever since. Karan has a keen interest in bone health, particularly in patients from various backgrounds, and he aspires to be an orthopaedic surgeon in the future. Karan hopes to maximise intensive research so that he can use evidence‐based medicine to assist his future patients and ultimately improve health outcomes.

Vanessa Gan is a non‐Indigenous research assistant working on various bone and muscle health projects. She is a recent graduate from Monash University and her work is predominantly assisting others in screening, imaging, and analysing bone and muscle scans.

Jesse Zanker is an Australian‐born man (he/him), descendent of European migrants and convicts, raised on the unceded lands of the Yorta Yorta people. He does not identify as an Aboriginal or Torres Strait Islander person. He recognises the history and continuing impact of colonisation on First Nations peoples, which informs his health equity approach to research. Through his training and work as a geriatrician and researcher working with First Nations peoples, he is committed to continually improving understanding of Indigenous Knowledges, which shape his co‐created research goals. He seeks guidance from those with experiences different to his own. He acknowledges the systems and structures which afford him unearned privilege.

Jennifer Browne is a non‐Indigenous researcher working predominantly in Aboriginal and Torres Strait Islander health. Her work is shaped by her background in public health and nutrition in the Aboriginal Community Controlled Health sector and an understanding of the historical, social, cultural and commercial factors influencing health outcomes for Aboriginal and Torres Strait Islander peoples.

Dr Jessica Bravo grew up in a small rural town in southern Ontario, Canada where she was heavily involved in local community activities and organisations. Coming from an immigrant family from South Africa, she moved to the city after high school to pursue a university education. After graduating from an Honours Bachelor of Science in Human Kinetics, she continued with her educational career, completing a Doctor of Chiropractic at the Canadian Memorial Chiropractic College, in Toronto, Ontario. Graduating with honours, on the Dean's list, Dr Bravo moved to Melbourne, Australia, to pursue her Chiropractic career. Shortly after the move, she became an Executive Director of Chiropractic Australia (CA) and opened her own practice in St Kilda, Victoria. With her focus being patient‐centred, evidence‐based care, she grew her clinic to three locations across Melbourne, while maintaining her role at CA and commencing a position as a Clinical Supervisor at RMIT University. Since the start of the COVID‐19 pandemic, Dr Bravo has continued her studies, completing a Master of Public Health, specialising in Chronic Disease and Indigenous Health. After eight years in the chiropractic profession, Dr Bravo has transitioned into a career in public health and now works at the Victorian ACCHO. Her focus, as a non‐Indigenous woman, is supporting a self‐determined workforce within ACCHOs across both Victoria and Australia, while also maintaining interest in chronic disease management and models of care.

Cat Shore‐Lorenti (they/them) is a Senior Clinical Trials Coordinator with Monash University, working and living on Bunurong Land. Specifically, their work is based at Monash Medical Centre Clayton, where more than two in five community members are from culturally and linguistically diverse backgrounds. Growing up hearing stories of their family members who survived war, religious vilification and forced displacement, who ultimately immigrated, initially to Tharawal/Dharawal Land in the 1960s, instilled a passion for and deep respect of diversity and inclusion as well as curiosity about other cultures. On another side of their family there are coloniser roots that were always acknowledged through a strongly left‐leaning political lens (advocating for access to First Nations cultural programs and educational workshops in high school, for example). They are always open to advice and suggestions on removing barriers to research projects and clinical trials for First Nations Peoples as well as people from culturally and linguistically diverse backgrounds.

David Scott is an Associate Professor at an Australian university with a diverse mix of domestic and international students, but only few Aboriginal and Torres Strait Islander staff and students. He is a first‐generation university graduate in exercise science who grew up in Tasmania, Australia, in a predominantly white community speaking English only. While living in Queensland for several years as an adult, he spent time as a football coach for a junior team with a number of Aboriginal and Torres Strait Islander children. His research focuses on reducing the burden of musculoskeletal health disorders in older people and is currently funded by a National Health and Medical Research Council Investigator Grant. His current and former research trainees include students from diverse backgrounds.

Jackson Baker is an accredited exercise physiologist who currently practises within the private sector in rural Victoria and NSW. Jackson has clinical experience in working within Aboriginal communities as an exercise physiologist and is currently involved in the chronic disease outreach program at Njernda Aboriginal Corporation. Jackson is passionate in regard to providing both adequate and high level health care to people living in remote and rural locations for overall greater health care outcomes.

Robin Daly is a non‐Indigenous man and Professor of Exercise and Ageing at Deakin University. His main area of research for the past 30 years has been to understand how exercise and nutritional approaches can prevent and manage chronic conditions, particularly musculoskeletal diseases, certain cancers, type 2 diabetes, and cognitive‐related disorders. His work has led to the implementation of evidence‐based, community exercise programs and nutritional products to optimise musculoskeletal health and body composition across a range of population groups and cultures.

### Ethics approval

In accordance with the *National statement on ethical conduct in human research 2007*,^16^, ^17^ the Monash Health Human Research Ethics Committee granted ethics approval (project number: RES‐19‐0000374A). We adhered to the Australian Institute of Aboriginal and Torres Strait Islander Studies’ *Code of Ethics for Aboriginal and Torres Strait Islander Research* ^18^ and the National Health and Medical Research Council's *Ethical conduct in research with Aboriginal and Torres Strait Islander peoples and communities: guidelines for researchers and stakeholders*.^19^

## Results

### Participant and focus group characteristics

Eighty‐two Indigenous people participated in the focus groups across 10 sites. Participants included Community members, Elders and Aboriginal health workers. Sixty‐four participants (78%) were women, and the majority lived in metropolitan centres, regional centres and large rural towns (Modified Monash categories 1–3). All participants lived in Victoria. Five participants (6%) had completed primary school only, 42 (51%) had completed high school, 17 (21%) had completed vocation education training and 14 (17%) had completed university education. The mean duration of the focus groups was 62 minutes (range, 46–97 minutes) (Box 3).

Box 3 **Characteristics of the 82 participants and the focus groups**

**Table:** 

Characteristic	Number of participants or focus group duration
Sex	
Men	18
Women	64
Age	
35–44 years	19
45–54 years	16
55–64 years	22
> 65 years	25
Education	
Primary school completed	5
High school completed	42
Vocation education training completed	17
University education completed	14
Missing data	4
Location	
Metropolitan (MM 1)	27
Regional centre (MM 2)	7
Large rural town (MM 3)	40
Medium rural town (MM 4)	8
Focus group duration (mean, 62 minutes)*	
Site 1	46 minutes
Site 2	83 minutes
Site 3	68 minutes
Site 4	95 minutes
Site 5	51 minutes
Site 6	62 minutes
Site 7	74 minutes
Site 8	75 minutes
Site 9	95 minutes
Site 10	97 minutes

MM = Modified Monash. * A total of 12 focus groups were conducted across 10 sites; two sites each held two focus groups and combined duration is shown for these.

### Themes generated

Five themes were generated from the data:

- knowledge of exercise for bone and muscle health;
- connection to Country;
- importance of regular preventive health activities;
- food and nutrients as good medicine for bone health; and
- healthy futures for Community through education.

Box 4 provides an overview of the major themes and subthemes. Participants shared similarities and insights across age bands and location by way of knowledge and perceptions on bone and muscle function in disease, and healthy translation with Community consideration. Major findings are reported under each framework category, including thematic title and important quotes reflecting participant lived experience.

### Ways of Knowing

Indigenous Ways of Knowing is an epistemological research method informing data analysis, drawing on knowledge of participants to illustrate ideas and perceptions.

The theme “knowledge of exercise for bone and muscle health” came through discussions around exercises considered most effective. Community conversations contrasted exercises most beneficial for bone and muscle and noted differences for exercises for cardiorespiratory health. Participants had mixed awareness about osteoporosis. Some were able to distinguish osteoporosis as a disease of bone, while other participants confused osteoporosis with osteoarthritis.

Relevant exercises for osteoporosis that participants mentioned included resistance training, running, jumping and landing sports such as tennis or netball. Some participants mentioned lower impact exercises such as swimming and cycling. Others acknowledged that higher intensities and loading were more beneficial for bone strength, including labour‐intensive physical activity.

The theme “connection to Country” was discussed by participants across sites. A key aspect of aligning Ways of Knowing with connectedness to Country was the concept of the land and its association with improved spiritual wellbeing and lifestyle. Participants emphasised that connection draws on traditional knowledge systems using ancestral remedies such as bush medicines and simultaneously requires careful adaptation for western medicine.

Participants openly expressed distrust of medical and western conventions, while a few expressed this same concern more covertly about certain medical topics that they perceived as detrimental for their health. Medications for bone health were acknowledged as playing a role, so long as lifestyle factors (good nutrition and supplementation), good mental health practices (including social connection, yarning and laughter) and physical activity were predominant and utilised before medications.

Notions of connection to Country were met with frustration by some older participants and Elders, expressing concerns about having access to — and proper use of — land to care for, utilising the land for healthy food growing, and having space for physical activity and spiritual connection (Box 4).

### Ways of Doing

Indigenous Ways of Doing informs data analysis, drawing on means and methods to become proactive within Community. The key themes identify proactivity within Communities.

The theme “importance of regular preventive health activities” emerged as participants noted the importance of general screening and raising Community awareness of bone and muscle health. Some participants had received appropriate screening and testing for osteoporosis and were aware that the best way to determine bone mineral density was through dual energy x‐ray absorptiometry (DXA) (Box 4).

Conversations among participants highlighted the interconnectedness of health conditions such as diabetes, heart disease, multiple sclerosis and muscular dystrophy, and their impact on bone and muscle health. Participants discussed how these conditions contribute to bone fragility and increase risk of fracture and falls, leading to a decreased quality of life. There was unanimous agreement on the importance of allied health as an essential service for bone, muscle and related lifestyle management, and the need for a multidisciplinary approach, providing holistic support to patients and considering Communities’ desires and needs.

Some discussions outlined the importance of multidisciplinary care in alignment with concerns about time constraints in seeing a general practitioner. Some participants felt neglected and not listened to. Concomitant lack of cultural awareness and competency training among health care professionals was reported.

Holistic health and its relationship with awareness and screening was also noted among participants, who discussed lifestyle factors and their relationship to musculoskeletal health. Examples included dental health, mental health, sound sleep, and connection with Community. There was less understanding regarding the association between physical organs (eg, heart or kidney) with bone and muscle health. Some participants, however, could make connections and discuss crossovers and relative significance through storytelling and lived experience.

The theme “food and nutrients as good medicine for bone health” was raised by participants. The importance of good nutrition and diet consisting of adequate nutrients including protein, calcium, magnesium and iron was brought up by participants across all sites. Participants confirmed that vitamin D was best sourced from sunlight over food, and that skin pigmentation played a role in absorption. They also noted higher Community susceptibility to iron deficiency.

Participants suggested that the best food sources supporting nutrient intake were traditional foods within the local context and participant's Country. Furthermore, participants noted that the introduction of some western foods, such as dairy, were beneficial for bone and muscle health (Box 4).

There was consistency among participants discussing that a foods‐first approach is better than dependence on supplements, and that supplements are useful in the absence of adequate nutrition. Dietary practices were emphasised as having to come from self‐determined Community decisions, discussed with collective consensus. While the importance of modern‐day food availability was considered, the practice of traditional ways with foods was emphasised by participants across all sites (Box 4).

Participants described how a downside of modern‐day foods was their unhealthy composition and impact on Indigenous health. This included available processed foods, such as those high in sugar, salt and fat. Participants noted that the current cost of living relative to their food budget, and physical access to foods depending on where participants lived, could perpetuate dietary deficiencies.

### Ways of Being

Indigenous Ways of Being is an epistemological research method that informs data analysis, reflected in the present actions and behaviours of participants’ life circumstances and how they can determine better future ways of being for Community.

The theme “healthy futures for Community through education” came through as all participants emphasised including past knowledges, skills and behaviours. Discussion of a “full circle” to incorporate modern‐day living with traditional Indigenous methods focused on nourishing the physical and mental health of Indigenous young people through to the Elders. A focus on these two life stages, through active and ongoing education, was emphasised across sites (Box 4).

Participants expressed concern about excess screen time among young people and recommended this as a key educational area to address. Revitalising traditional outdoor activities and practices that encourage physical activity was recommended. Furthermore, participants stated that visual and kinaesthetic aids using videos, posters and Community‐delivered demonstrations, such as group exercise or cooking classes, would enhance health education about lifestyle approaches for bone and muscle health and their relationship to falls risk (Box 4).

Local Community‐led education was preferred by participants. Participants explained that keeping ideas local created familiarity among group members and was considered more convenient and accessible for more local Indigenous Communities, especially in association with mental health practice and awareness. Many participants noted that these approaches encompassed holistic aspects of Indigenous Ways of Knowing, Being and Doing, and promoted social connectedness, active engagement, self‐determination and a sense of belonging (Box 4).

Box 4 **Themes and subthemes from Community participants**

**Table:** 

Theme or subtheme	Community voice
Health prevention through screening and Community awareness — the importance of lifestyle	“It usually starts through nutrition in our mothers, what mum's taking into breastfeeding and if it's healthy or not and [our bones and muscles are healthier] when thinking about our mental health … ensuring we got adequate housing and you know finances.” (female, regional)
	“All the stuff that you’re talking about, is they may lose all that density in bone … x‐ray picks it [osteoporosis] up but I’ve had one of those other scans [DXA] that does your bones properly.” (male, regional)
Healthy futures for Community through education and connection to Country	“It's also about education too, what you’ve been brought up with from the past Aboriginal people. Yeah. But I’d say what we used to do, you know, what's natural medication that's non‐pharmacological.” (female, regional)
	“Yeah and I also want to add that we don’t have access to land. Some of us do and it might be a small portion. Traditionally what our people do is we move around our Country due to what is in season at the time. So we’d never take anything more or take only what was needed … our soil was very very good, we had inches of it, now we only have a little bit … and we would get a lot of iron and nutrients because we cooked in the ground.” (female, metropolitan)
	“We [the Community] need to get recognition of our ways of dealing with things too, like grief … There's a lot of the pills, medications and things like that, but there's nothing much else to deal with. You know a big thing of mental health and spiritual and emotional wellbeing is not, in my opinion, being looked at as part of healing for our Communities. And I do believe that it's just dealing with the symptoms, and not with the cause of everything that we’re going through with our Community.” (female, regional)
	“I definitely think showing [resistance training] technique is important and how to do it correctly [for Elders] so you don’t injure yourself. I mean I don’t know if they would just get up and put resistance bands on and squat … but those exercises they can do sitting with the resistance bands.” (female, metropolitan)
	“We go back to that whole thing of you’ve got to have a good GP and access to health services. There should be more, you know, workshops around health. We don’t do enough for Aboriginal people and today we’ve had a lot of [bone and muscle] information where we [previously] thought we couldn’t get any … There should be a youth session, a mid‐life session and an Elder session.” (male, regional)
Knowledge of exercise for bone health	“[With our bones] exercise goes with mental health and it helps your mental health … Like it's a cycle. When your mental health is good you want to exercise.” (female, metropolitan)
	“The last 3 months, I’ve had a wheelbarrow full of heavy wood and I’m coming up the hill, and I have to come up the ramp, put it on the back veranda.” (female, regional)
Food and nutrients as good medicine for bone health	“It's the same if I don’t take the vitamin D. Why are my bones bloody aching? So that's [helped] part of my night‐time routine now, yeah.” (female, metropolitan)
	“Let's talk about calcium, it's milk, cheese, yoghurt, all the dairy products, green leafy vegetables and everything that you’ve got to eat properly.” (female, metropolitan)
	“In order to have good bone structure, we need to get out of the [modern‐day] foods we eat and get more plant‐based foods, we need to get zinc, we need to get potassium, calcium, vitamin A and D … so we have the strength in those bones to do the things that you wanna do.” (female, regional)
	“Your mental health needs to come into here. Because if you’re mentally not okay, it's hard to get out of here to get it [exercise]. Then, the other thing I would add in regard to that would be sleep and financial stability. Because financial stability comes into the diet and nutrition, if you don’t got money you can’t go buy your fancy salads every day and your veggies and all that.” (female, metropolitan)

GP = general practitioner.

## Discussion

Our key findings show that participants are aware of the importance of exercise for bone health. Most participants demonstrated an understanding of nutrition in bone health, with a focus on a healthy diet first and supplements if necessary. Holistic health, including connection to Country, preventive screening and raising awareness, was discussed and other lifestyle factors and their relationship to musculoskeletal health were highlighted. Increasing health literacy among Community with targeted education programs using broader health promotion awareness was valued and considered beneficial across the life course.

Participants acknowledged that high intensity and loading exercises improved musculoskeletal health. Studies have shown that resistance and impact training (eg, weightlifting or sports such as tennis and netball) increase bone mineral density and muscle strength compared with cardiovascular exercises (eg, swimming or cycling).^20^, ^21^ This contends with prior research, suggesting some perceptions of exercise being considered painful, or that pain is not discussed.^22^, ^23^, ^24^

While there is recognition of the importance of physical activity within Indigenous Communities, there are barriers to participation. For instance, lack of access to transport and resources may explain lower rates of participation in sports and exercise.^22^, ^23^, ^25^ More recently, physical activity promotion to address falls risk in Aboriginal people aged > 45 years has been embedded and well received by local ACCHOs.^26^ A systematic review of Indigenous peoples’ perceptions about physical activity showed that lack of resources and living in rural areas hinder involvement in physical activities.^23^ Although not focused on bone‐specific exercises, the Aboriginal and Torres Strait Islander Women's Fitness Program was a structured, progressive 12‐week group program aimed at reducing waist circumference and improving metabolic health in an urban setting.^27^ A mixed methods process evaluation of the trial showed those who struggled to attend classes experienced several competing obligations, or had a major event occur, such as a death in the family.^28^ We recommend ongoing financial and government support for health promotion programs incorporating supportive environments, redirection of health care services, and involvement of Aboriginal health services at the forefront of these initiatives to offset barriers and contribute to effective delivery and approach most suited to the Community.

In our study, diet was regarded as important to maintain bone health. Calcium, vitamin D and respective food sources were identified as significant nutrients for bone health. In a systematic review evaluating the effectiveness of nutrition education in improving nutrition‐related health outcomes among Indigenous people, it was reported that the components that had greatest impact were cooking workshops, group education sessions and store interventions.^29^ Other contributing factors included start‐to‐finish involvement of Community implementation, Aboriginal health worker involvement, environmental changes and improved access to health care.^29^ In another systematic review, it was reported that traditional foods were valued by connecting to culture and improving nutrition, although access was limited.^30^ Consistent with this, we found that participant knowledge and practice using traditional foods were paramount. Food and nutrition knowledge and skills are important factors influencing diets. Studies have found that participants seek to increase confidence in preparing healthy, simple and affordable family meals.^31^, ^32^, ^33^, ^34^ Furthermore, practical, Community‐directed nutrition education programs are effective in improving dietary intake and health behaviour.^29^, ^35^ Various obstacles, such as poor education around diet, inaccessibility to nutrient‐rich foods, increased cost of living and expense of healthy foods, require stronger public health initiatives.^36^ These are barriers to maintaining and sustaining a balanced diet, and they jeopardise the health outcomes of Indigenous people — consequently these have a knock‐on effect as they increase the likelihood of chronic diseases, such as osteoporosis.

Regular health screening and increasing Community awareness about musculoskeletal health was an important theme raised by participants. Accessing health care services is crucial to maintaining musculoskeletal health, especially for Indigenous people who experience higher rates of falls and fractures.^37^, ^38^ However, Indigenous people often struggle to access these services owing to a range of factors, such as high cost of care, poor communication, lack of cultural safety, and racism from health professionals.^38^, ^39^ Strategies to overcome these barriers include tailoring and managing health care services through self‐determination. ACCHOs demonstrate improvements in primary health care within the Community. Indigenous people are more likely to engage in preventive health behaviour, including regular screening and awareness campaigns aimed at musculoskeletal health, if there is Community ownership and involvement in health promotion. Furthermore, connection to Country integrates spending time exercising outdoors, together, and promotes added benefits through green exercises. Incorporating Indigenous perspectives into health care strategies is crucial for addressing the unique health needs of Indigenous people. In addition, integration of diagnostic tools such as DXA to assess bone mineral density identifies individuals who have or are at risk of osteoporosis, enabling targeted interventions to improve overall bone health outcomes. Thus, provision of care and ongoing collaboration with holistic health care concepts within Indigenous Communities is likely to be more effective.

### Strengths

A significant strength of our study was that the research was led by an Aboriginal researcher and clinician from Victoria who has qualifications and experience in nutrition and musculoskeletal health. This allowed for a large sample size in a qualitative study and permitted development of a health professional and Community member Aboriginal advisory board. Furthermore, focus groups enabled diverse perspectives and discussions about musculoskeletal health among Indigenous people, adding to an area of Indigenous health that has scarce data. This approach, of understanding needs through feedback from many Community representatives across different regions within many different lands and nation groups, yielded valuable insights into the Communities’ perspectives on musculoskeletal health.

### Limitations

A limitation of our study was that it was halted due to the COVID‐19 pandemic; however, this provided the lead Aboriginal researcher with more time to recruit participants. Also, Indigenous men were relatively under‐represented. We recognise that the themes generated from our focus groups may not fully reflect the breadth of perspectives of the broader Indigenous Community. To address this, future research could explore a multimethod approach such as conducting surveys across a larger number of participants and in more regions of Australia, to ensure comprehensive representations of perspectives and experiences of Indigenous people.

### Conclusion

Holistic health considering multiple lifestyle factors and their relationship to musculoskeletal health was highlighted among Indigenous people across Victoria. There are several barriers to improving musculoskeletal health, including a key component of connection to Country, which is often neglected in Indigenous people's health. Increasing musculoskeletal health literacy by incorporating co‐created Community education, embedded in principles of health promotion, was valued by Indigenous people across the life course. To be effective, incorporating traditional Indigenous Ways of Knowing, Doing and Being with present‐day health evidence is required.

#### Data sharing

The data for this study will not be shared, as we do not have permission from the participants or ethics approval to do so.

## Competing interests

No relevant disclosures.

Received 2 July 2024, accepted 18 December 2024

## Supporting information


Supplementary tables
