# Supplementary material for: Bone health perspectives among Indigenous people: a qualitative study
Source: Med J Aust. 2025 Jun 17;223(2):92–100. doi: 10.5694/mja2.52704 (PMC12276726; doi:10.5694/mja2.52704)
Supplement: Supplementary file 1 — Supplementary tables [file MJA2-223-92-s001.pdf]

Supporting Information

Supplementary material

This appendix was part of the submitted manuscript and has been peer reviewed. It is posted as supplied by the authors.

Appendix to: Walker T (Yorta Yorta), Singh KP, Gan V, et al. Bone health perspectives among Indigenous people: a qualitative study. *Med J Aust* 2025; doi: 10.5694/mja2.52704.

Abbreviations

ACCHO: Aboriginal Community Controlled Health Organisation

AAL: Aborigines Advancement League

BDAC: Bendigo & District Aboriginal Co-operative

LHD: Local Health Districts

NAC: Njernda Aboriginal Corporation

RAC: Rumbalara Aboriginal Co-operative

VAHS: Victorian Aboriginal Health Service

Box 1. CONSIDER statement

**Domain**	**SIMBA Research Project**
**Governance**	
1. Describe partnership agreements between the research institution and Indigenous-governing organisation for the research (e.g., Informal agreements through to Memorandum of Understanding or Memorandum of Agreement)	- Study was led by Aboriginal researcher and clinician who made informal agreements with multiple (8) ACCHOs (Apr 2021-Sept 2023) across Victoria, particularly Aboriginal Health Workers that the lead researcher knew or had connections with<br>- All agreements were made via yarning and in-person visits across ACCHOs, phone calls, online meetings and emailing
2. Describe accountability and review mechanisms within the partnership agreement that addresses harm minimisation	- Monash Health Human Research Ethics Committee (HREC) approval with adherence to the AIATSIS *Code of Ethics for Aboriginal and Torres Strait Islander Research* and the *Ethical conduct in research with Aboriginal and Torres Strait Islander Peoples and Communities: guidelines for researchers and stakeholders* for the project was the first step in addressing harm minimisation<br>- An Aboriginal Advisory Group was developed with five members representing multiple ACCHOs and/or having direct involvement with Aboriginal and Torres Strait Islander health research and practice
3. Specify how the research partnership agreement includes protection of Indigenous intellectual property and knowledge arising from the research, including financial and intellectual benefits generated (e.g., Development of traditional medicines for commercial purposes or supporting the Indigenous Community to develop commercialisation proposals generated from the research)	- The partnership agreement was informally manifest, all aspects of this research included informed consent and ongoing guidance from ACCHO contacts known to the first author and were fortified with added counsel from the Aboriginal Advisory Group<br>- All involved participants as well as the ACCHO health workers were remunerated with monetary incomes or gift cards<br>- Feedback from participant input was ongoing with health workers via online meetings and phone calls, the data collected throughout the study was co-created with ongoing input and cross-checked with the Advisory Group and all module education developed thereafter was based on a train-the-trainer model where ACCHO health workers were able to make the content their own for their respective Elders and Community interests
**Prioritisation**	
4. Explain how the research aims emerged out of priorities identified by either Indigenous stakeholders, governing bodies, funders, non-government organisation(s), consumers and empirical evidence	- Community voice around bone health and its relationships to chronic disease and social and emotional wellbeing were not well known, researched or understood prior to commencing this research. Perceptions, values and beliefs around holistic health practices for bone health awareness required Community input from multiple ACCHOs around the state<br>- Earlier research done by members of this research group showed that bone health, falls and related fracture risk were scarce and that most data was quantitative and did not have a qualitative component required to better understand what Aboriginal health Workers (AHWs) and Community valued [1,2]<br>- Health promotion efforts have been extensively raised in the form of cognitive decline, dementia awareness and the associated risk of falls and injuries in Victoria's peak health body (VACCHO) and through a recent Royal Commission into My Aged Care impacting Indigenous Elders. This data indirectly builds on this and establishes the importance of social and emotional wellbeing and solidification of holistic approaches for bone health awareness
**Relationships (Indigenous stakeholders/participants and Research team)**	
5. Specify measures that adhere and honour Indigenous ethical guidelines, processes and approvals for all relevant Indigenous stakeholders, recognising that multiple Indigenous partners may be involved, e.g., Indigenous	- Victoria presently has no formal process for Aboriginal ethics approval<br>- Informal and localised ACCHO processes were utilised by lead Aboriginal investigator to ensure contextual cultural practices, as well as connecting with other ACCHO

ethics committee approval, regional/national ethics approval processes	members who introduced us to other Community workers and Elders who they recommended or advised would be keen to participate<br>- The Aboriginal Advisory Group gave further ideas and suggestions to ensure processes were respectfully met and abided by in their areas and some made recommendations to connect the lead Aboriginal investigator with more Community
6. Report how Indigenous stakeholders were involved in the research processes (i.e., research design, funding, implementation, analysis, dissemination/recruitment)	- Project design was initially agreed upon by two local Aboriginal organisations (local to the lead Aboriginal investigator) and snowballed with consensus agreement following discussion and conversations about the project; further input came from Aboriginal Advisors working in clinical practice (VAHS) and another working in research (University of Melbourne)<br>- The peak health body in Victoria (VACCHO) also had informal discussions to assist in recruitment and findings dissemination with lead Aboriginal investigator as a part of other extended Aboriginal health projects he was involved in independent of this work<br>- Research design was cross-examined by the Aboriginal Advisory Group and implementation was done with ongoing (pre and post) online meetings with local ACCHOs involved across Victoria (NAC, RAC, BDAC and AAL)
7. Describe the expertise of the research team in Indigenous health and research	- The Aboriginal Advisory Group has past and present clinical and research experience in Aboriginal health across musculoskeletal management<br>- The lead Aboriginal investigator has 15 years of clinical experience with 12 years spent working with ACCHOs in practice and 7 years' experience in Indigenous health research across public health, nutrition, age-related muscle loss and bone health<br>- Extended collaboration included guidance and counsel from two experienced Indigenous public health researchers who are very well respected and known in their field
**Methodologies**	
8. Describe the methodological approach of the research including a rationale of methods used and implication for Indigenous stakeholders, e.g., privacy and confidentiality (individual and collective)	- The Indigenous framework by Karen Martin-Booran Mirraboopa was applied and is grounded on three concepts: Ways of Knowing, Ways of Being and Ways of Doing. This method has been used in past Aboriginal research by the lead Aboriginal investigator and effectively helps theme Indigenous voices based on a non-Western methodology<br>- Yarning circles within ACCHOs (focus groups) were run with participants across Victoria and some sites had multiple sessions to help balance and ensure Community representation in instances where some organisations included lots of staff members<br>- All participants were de-identified and not named in any of the research project, a record of age bands across nine-year intervals was used to assist in the association with bone health and ageing<br>- The ACCHO feedback after each yarning circle at respective organisations was well favoured both by individuals and groups for knowledge and education and ongoing opportunities were mentioned and provided by both Elders and Community members to come back and help raise more health promoting ideas around this topic
9. Describe how the research methodology incorporated consideration of the physical, social, cultural and economic environment of the participants and prospective participants (e.g., impacts of colonisation, racism, social justice), as well as Indigenous worldviews	- Study design ensured that participant engagement across ACCHOs was flexible for all participants, particularly Elders, as well as being ongoing with liaison from Aboriginal health and Elder care workers; meetings and engagements were held at times befitting the ACCHOs so as to not impede with any local cultural business<br>- Yarning circles were selected by lead Aboriginal investigator following conversations with each ACCHO to better understand all worldviews from participants and create a sense of cultural appropriateness when the

	Aboriginal investigator was not local to the contextual yarning circles held in different lands and nation groups<br>- The Aboriginal Advisory Group continued to provide ongoing advice on considering broader social health determinants when organising localised yarning circles to consider needs of those who may not have ready access to direct services (i.e. transport, meals provided for, gift cards provided to local Aboriginal health workers aiding and assisting the lead investigator with recruitment for participation)
**Participation**	
10. Specify how individual and collective consent was sought to conduct future analysis on collected samples and data (e.g., Additional secondary analysis; third parties accessing samples, tissues, genetic, blood for further analysis)	- No biological analysis was done in this aspect of the research project<br>- Data was collected and then protected following a plain language statement with informed consent to participate in the yarning circles (focus groups); only the research team have access to this data and all of it is encrypted and password protected as well as de-identified<br>- Follow-up with the option to withdraw and cease use of data following focus groups was announced prior to and afterwards with participants themselves (prior to) and the ACCHO liaison or contact people for the lead Aboriginal investigator
11. Describe how the resource demands (current and future) placed on Indigenous participants and communities involved in the research were identified and agreed upon including any resourcing for participation, knowledge, and expertise	- The Aboriginal lead investigator was conscientious to ensure to practice cultural (colonial) load as a concept with himself and the extended Aboriginal participants and team members as a part of the advisory committee<br>- All participants were offered a multi-purpose credit ($50) for their time and contributions and those who were interested in becoming facilitators at their local ACCHOs were remunerated with an income granting permissions from their current working role
12. Specify how biological tissue and other samples including data were stored, explaining the processes of removal from traditional lands, if done, and of disposal	- This was not a component of this research study
**Capacity**	
13. Explain how the research supported the development and maintenance of Indigenous research capacity (e.g., specific funding of Indigenous researchers)	- The conception of the project considered and provided funding for a co-lead Aboriginal researcher to support the grant writer and other researchers<br>- Funding was provided by the Monash National Centre for Healthy Ageing (NCHA) [no grant number].<br>- Other capacity building was possible to train local Aboriginal Health Workers in ACCHOs by our lead Aboriginal investigator and other non-Aboriginal investigator in the domain of bone health and Community themes arising from the yarning circles with Elders. The Aboriginal Health Workers were able to be paid for their training time to deliver to the Community how they best felt culturally appropriate
14. Discuss how the research team undertook professional development opportunities to develop the capacity to partner with Indigenous stakeholders?	- This process was largely informal and naturalistic. The team undertook regular online meet-ups and in addition our lead Aboriginal investigator met and spoke regularly with our Advisory Group and key contacts at ACCHOs who were keen to continue learning about the project and the research developments; this could then be taken and translated to the other members of the research team and used to refine better ways of Indigenous engagement as the project evolved<br>- Another training session was organised by lead Aboriginal investigator early (1 May 2021) with a local Aboriginal organisation he works with to help educate around local (Yorta Yorta) customs in considering research for the broader research team that were non-Indigenous
**Analysis and interpretation**	
15. Specify how the research analysis and reporting supported critical inquiry and a strength-based approach that was inclusive of Indigenous values	- Involvement and contribution from ACCHO stakeholders assisted ongoingly with analysis and reporting based on what was discussed. There was also pre and post focus group yarning with Community leaders at each respective

	site to ensure best disaggregated practice for context of each respective Community involved in the project<br>- While the nuances of deficit discourse in certain topics were noted, the questions and framing were led by both our Aboriginal lead and Aboriginal Health Workers at each site to ensure language and discussion was strength-based in driving participation and to properly reflect Indigenous values across all sites
**Dissemination**	
16. Describe the dissemination of the research findings to relevant Indigenous governing bodies and peoples	- Findings within the primary research were dynamically themed and then immediately utilised with each respective ACCHO involved shortly after. This was done by way of ongoing education and train-the-trainer content, often delivered by a local Aboriginal Health Worker or Elder/Aged Care Worker<br>- This manuscript will permit for Aboriginal and Allyship to build upon the scarce space in Aboriginal bone health and give Aboriginal lead author the opportunity to disseminate this knowledge at Aboriginal conferences, workshops and related areas (lunch and learns [feed and a cuppa] within ACCHOs and other Community areas) directly relevant to peak health Aboriginal bodies to build greater ACCHO autonomy and use when and as required
17. Discuss the process for knowledge translation and implementation to support Indigenous advancement (e.g., research capacity, policy, investment)	- All content developed will be available for trainers and educators within each respective ACCHO. Healthy Bones Australia have aligned with us to ensure broader advocacy of this content online with our Aboriginal lead author<br>- The Aboriginal lead investigator has also provided his contact information for additional online or phone sessions when or if needed by the trained facilitators for the education content and results of this research<br>- Successful publication in a reputable journal within Australia will help the lead investigators to further apply for essential funding to improve the scope and awareness of bone and muscle health amongst Aboriginal people

Box 2. SIMBA: focus group questions

Facilitator introduces themselves and the observer. Then asks everyone to introduce themselves one by one (name and where they're from). Following introductions, Facilitator will announce the commencement of recording of the focus group session (details will be in consent form before focus group session). Facilitator reads introduction then asks topic questions and prompts as needed.

Study Introduction

It's important to look after our bones and muscles, to make sure we can move around freely without any help. SIMBA is the Study of Muscle and Bone Ageing in Indigenous Australians. With this focus group session, we would like to work together with you all, and develop a community muscle and bone health education program. Our aim is to improve knowledge about bone and muscle health in Aboriginal and Torres Strait Islander adults. In this focus group session, we're first going to talk about bones, then we'll talk about muscles.

Section 1: Bone Introduction

Our bones support us and allow us to move around and maintain independence. Our bones store minerals for example calcium, which help keep our bones strong. There are many things we can do to keep our bones healthy and strong. Similarly, there are things that we do that put us at a greater risk of breaking a bone (having a fracture). If we don't eat and drink foods rich in calcium and don't do enough bone-building exercise, our bones can become weak, like a honeycomb, and then break. Broken bones (called fractures) can be painful and sometimes need surgery to heal. They can also cause long-lasting health problems.

1. What do you think is important for your bones to stay strong and healthy?

Prompt:

- Do you think exercise is important?
- What types of exercise do you think would be best for your bones?
- What about diet and nutrition? (calcium, vitamin D and protein)

2. What do you know about "osteoporosis"?

Prompt:

- When bones lose minerals like calcium more quickly than the body can replace them, this leads to a loss of bone strength (or density). With osteoporosis, bones become thinner and in severe cases, even a minor trip can cause bones to break.
- How do you know if you have osteoporosis?
- What might cause a bone to break (fracture)?
- Do young and old or men and women have a similar risk of having osteoporosis?

Section 2: Muscles Introduction

Having strong muscles is also important so that we can move around without any help. When you make your muscles work by being active or doing exercises, they grow stronger. If you have better muscles as an adult, it will help prevent you from falling!

3. In your own words, can you explain any other long-term diseases that may also affect bone and muscle health?

Prompt:

- For instance, how about diabetes, heart or kidney disease?
- How would any of these diseases affect your chances of falling?

4. Now I want you to imagine that you have long-term disease and you just fractured or broke your hip. It turns out that because of your condition, your bones aren't as strong, and your muscles are weak. What would you say to your specialist?

Prompt:

- How do you think learning more can help in these situations?

Section 3: Practicalities

5. Please rank the following topics in order of importance for muscle and bone health? Use the strips of paper for this exercise

6. What other topics related to bone/muscle do you think are important and should be included? Allow group to brainstorm and write down new ideas on the blank strips and ask to re-order strips if necessary.

7. Given this list of important things to know about your bones and muscles, in what ways can this information be provided?

Prompt:

- What about face-to-face seminars in a group setting? Or videos available on a website?
- If seminars: how would you find this most effectively delivered? E.g. informal round table discussion, using a PowerPoint presentation, etc
- How useful would you find information sheets that summarise what was discussed during each session?
- What time of day would be most suitable?
- What locations would be most suitable?
- Who should attend these? Should there be separate ones held for particular groups?

References

1. Zengin A, Shore-Lorenti C, Sim M, Maple-Brown L, Brennan-Olsen SL, Lewis JR, Ockwell J, Walker T, Scott D, Ebeling P. Why Aboriginal and Torres Strait Islander Australians fall and fracture: the codesigned Study of Indigenous Muscle and Bone Ageing (SIMBA) protocol. *BMJ Open* 2022 Apr 4; 12(4): e056589.
2. Zengin A, Maple-Brown LJ, Brennan-Olsen S, Center JR, Eades S, Ebeling PR. Musculoskeletal health of Indigenous Australians. *Archives of Osteoporosis* 2018 Jul 14; 13(1): 77.
